# Stochastic Amplification of Fluctuations in Cortical Up-States

**DOI:** 10.1371/journal.pone.0040710

**Published:** 2012-08-07

**Authors:** Jorge Hidalgo, Luís F. Seoane, Jesús M. Cortés, Miguel A. Muñoz

**Affiliations:** 1 Departamento de Electromagnetismo y Física de la Materia e Instituto de Física Teórica y Computacional Carlos I. Universidad de Granada, Granada, Spain; 2 Bernstein Center for Computational Neuroscience, Technische Universität Berlin, Berlin, Germany; 3 Institut de Biologia Evolutiva, UPF-CSIC, Barcelona, Spain; 4 Departamento de Ciencias de la Computacion e Inteligencia Artificial, Universidad de Granada, Granada, Spain; National Research and Technology Council, Argentina

## Abstract

Cortical neurons are bistable; as a consequence their local field potentials can fluctuate between quiescent and active states, generating slow 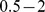 Hz oscillations which are widely known as transitions between Up and Down States. Despite a large number of studies on Up-Down transitions, deciphering its nature, mechanisms and function are still today challenging tasks. In this paper we focus on recent experimental evidence, showing that a class of spontaneous oscillations can emerge within the Up states. In particular, a non-trivial peak around 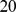 Hz appears in their associated power-spectra, what produces an enhancement of the activity power for higher frequencies (in the 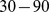 Hz band). Moreover, this rhythm within Ups seems to be an emergent or collective phenomenon given that individual neurons do not lock to it as they remain mostly unsynchronized. Remarkably, similar oscillations (and the concomitant peak in the spectrum) do not appear in the Down states. Here we shed light on these findings by using different computational models for the dynamics of cortical networks in presence of different levels of physiological complexity. Our conclusion, supported by both theory and simulations, is that the collective phenomenon of “stochastic amplification of fluctuations” – previously described in other contexts such as Ecology and Epidemiology – explains in an elegant and parsimonious manner, beyond model-dependent details, this extra-rhythm emerging only in the Up states but not in the Downs.

## Introduction

The cerebral cortex exhibits spontaneous activity even in the absence of external stimuli. Deciphering its oscillations and their correlates to behavior and function are major challenges in Neuroscience [1], [2]. Thus, for instance, high-frequency neural activity in the 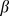 and 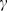 ranges (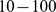 Hz) has been related to a plethora of cognitive tasks including action, perception, memory, or attention [1]. On the other hand, slow 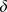 waves (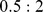 Hz) are preponderant during the deepest stages of sleep, under anesthesia, or during quiet wakefulness [3]–[5], and may play an important role in neural plasticity and in the consolidation of new memories [6]. Finally, changes in the pattern of global activity are associated with brain-state transitions such as sleep-wake or to pathologies such as epilepsy [7]. Remarkably, very similar patterns of activity have been observed *in vitro* as well; both, coherent oscillations in the beta-gamma ranges and slow oscillations have been reported in brain slices [8]–[11], what suggests that these spontaneous oscillations are intrinsic to the dynamics of cortical networks.

These slow oscillations appear in the form of *Up-and-Down states* in which a large fraction of neurons alternate coherently between two different stable membrane-potential states: the quiescent *Down state* –with a high degree of hyper-polarization and very low activity– and the depolarized *Up state* –with high synaptic and spiking activity– [12]. The coherent (though non-periodic) -alternation between Up- and Down- states gives rise to Up-and-Down transitions, resulting in low-frequency 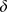 waves [13]. The function and role of such transitions at the global network level are not fully understood (see [14] and references therein). The origin of such a bistability in the cortex dynamics has been argued to rely either on intrinsic neuronal features [9], [15], [16] or on network-level properties [17]–[19]. Even if its nature is not universally agreed upon, most of the existing computational models for cortical Up-and-Down states feature network rather than cellular mechanisms [13]. Here, we will focus on network models in which the cortex bistability emerges as a collective network phenomenon.

Existing computational models for network bistability involved some regulatory mechanism such as short time synaptic depression [18], [20], [21] or the presence of inhibitory populations of neurons [16], [17], [22]. Any of these ingredients (repressors) provides a negative feedback mechanism able to control the overall level of activity generated by self-excitation, allowing for the network to self-regulate. Generically, network models including activator/repressor dynamics may exhibit two different possible outputs, with low and high levels of activity, respectively. Although it is also possible to switch in the absence of noise between these two levels (eg. through a limit cycle), most of the previous models incorporate noise-induced Up-Down transitions, and in this paper we follow this strategy.

Given the apparent dichotomy between slow and high-frequency oscillations and their distinct cognitive correlates and function, the empirical finding that slow and fast rhythms may coexist might sound surprising but it has been shown to occur by different authors. Firstly, Steriade et al. found that high-frequency oscillations occurred within the active intervals of slow oscillations [23]. In similar experiments, Mukovski et al. [24], Fujisawa et al. [25], and more recently Compte and coauthors [26] have shown that high-frequency oscillations –in the 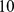
–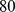 Hz range– develop within the Up intervals of Up-and-Down states. In particular, the power spectrum of such oscillations develops a pronounced peak at some frequency in the 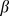-band –between 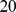 and 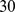 Hz– together with a substantial increase in the spectral power all along the 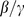 range. Remarkably, no similar peak has ever been observed in Down states [25], [26].

Another remark acknowledged by Compte et at. in [26] is that, while measurements of local field potentials in the Up state reveal robust oscillations in the 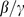, individual membrane potentials at the intracellular level do not show any trace of similar oscillations in that frequency band. This suggests, on the one hand, that high-frequency oscillations are a collective phenomenon emerging at the network level and, second, that there is no global synchronization (frequency locking) of individual neurons to the systemic rhythm. Thus, individual neural rhythms and the global emerging rhythm are independent.

At the modeling side, several authors have before addressed some of these issues and computed, in particular, the power-spectrum of network oscillations. For instance, Kang et al. [27] studied a mean field model in the presence of noise. They performed an analytical calculation of the power spectrum of a Wilson-Cowan-like model with excitatory and inhibitory neurons and showed the emergence of a resonant peak at gamma frequency. In a similar model, Wallace et al. [28] made the noise variance to scale with the network size and derived analytically the power-spectrum showing that it is possible to have coexistence of high-frequency oscillations for the population without having oscillations for individual neurons. On the other hand, for spiking neural networks, Spiridon and Gerstner [29] showed that the noise accounting for network-size effects affected the power-spectrum of the population activity. Similarly, and by using a Fokker-Planck formalism, Mattia and Del Giudice [30], [31], described the time evolution of the average network activity in presence of size-effects noise, and analytically derived its power spectrum and their resonant peaks.

Even if much has been written and is known about neural oscillations, our goal here is to shed some more light on the previously discussed questions by studying general aspects, beyond modeling details, as well as a simple and general theory accounting in general for the above described phenomenology and, in particular, for the asymmetry between Up state and Down state power spectra. For this, we study two different network models, one mean field and the other a network of spiking neurons, and discern whether high-frequency collective oscillations exist within the Up and/or within the Down state, respectively. Some of our results coincide with existing ones, as those reported in the previous paragraph, but, using a unified approach, here we conclude that a phenomenon termed *stochastic amplification of fluctuations* which can operate during Up –but not Down– states explains all the observations above in a robust, precise, and parsimonious way.

## Materials and Methods

Hereafter, we present two different network models reproducing the dynamics of Up-and-Down states, one based on a mean-field single population model (Model A) and one based on a network of spiking-neurons (Model B). Our strategy is to keep models as simple as possible to uncover the essence of Up-and-Down states. The theory of stochastic amplification of fluctuations, aimed at accounting for the non-trivial phenomenology above beyond modeling details, is presented also in this section.

### Model A: Minimal model for Up-and-Down states

The simplest possible models for Up and Down states have a deterministic dynamics and characterize neural network activity by a global (“mean-field”) variable, the population averaged firing rate (which is a proxy for measurements of local field potential). Different models including synaptic depression and/or some other regulatory mechanism such as inhibition, have been employed in the past to describe Up and Down states. We focus here on the model proposed by Tsodyks *et al.* [32], [33]) including activity-dependent short-term synaptic plasticity as the key regulatory mechanism. In the Appendix S1 we present results for a similar model with inhibition. In this context, Up and Down states correspond to fixed points of the deterministic dynamics with, respectively, high and low firing-rates. The deterministic model is described by the mean membrane potential, 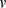, and the variable 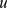 accounting for the strength of synaptic depression. This second variable mimics the amount of available resources (varying between 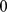 and 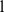) in the presynaptic terminal to be released after presynaptic stimulation, thus, the larger 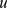 the more synaptic input arriving to the postsynaptic cell [32], [33]. The mean voltage grows owing to both external and internal inputs and decreases owing to voltage leakage. On the other hand, synaptic resources are consumed in the process of transmitting information and generating internal activity (providing a self-regulatory mechanism) and spontaneously recover to a target maximum value, fixed here to 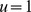:


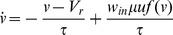



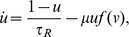
(1)

where 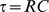 (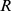 membrane resistance and 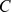 capacitance) and 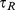 are the characteristic times of voltage leakage and synaptic recovery, respectively, 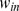 is the amplitude of internal inputs, 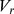 is the resting potential, and 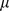 is the release fraction indicating the efficiency of synapses. The firing rate function, 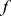, is assumed to depend on 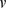 as 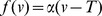 if 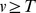, where 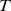 is a threshold value, and 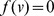 otherwise (i.e. it is a “threshold-linear” gain function). External inputs could also be added to the model, but they are irrelevant for our purposes here. Spontaneous transitions between these two stable states can also be described within this framework by switching-on some stochasticity. Possible sources of noise are network size effects, sparse connectivity, unreliable synaptic connections, background net activity, synapses heterogeneity, or irregular external inputs. An instance of this stochastic approach is the work of Holcman and Tsodyks [18] (see also [34]) where a noise term was introduced into the above mentioned mean-field model with synaptic depression. Indeed, adding uncorrelated Gaussian white noises, 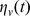 and 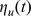, of amplitude 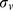 and 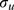 respectively, to equation 1, converts them into a set of stochastic/Langevin equations [18]. While the noiseless version of the model presents bistability its noisy counterpart exhibits Up-and-Down states.

### Model B: Spiking-neuron network model for Up-and-Down states

Millman and coauthors [21] proposed an integrate-and-fire (neuron-level) generalization of the model above, including some additional realistic factors. These refinements allow us to compare the emerging results with empirical ones not only qualitatively but also quantitatively. The model (Model B, from now on) consists in a population of 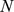 leaky integrate-and-fire neurons, each one connected by excitatory synapses with (on average) another 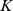 of them, forming a random (Erdos-Renyi) network. Each neuron is described by a dynamical equation for its membrane potential 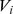 (with 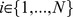) in which 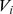 increases owing to (i) external (stochastic) Poisson-distributed inputs arriving at rate 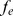 and (ii) internal inputs from connected spiking pre-synaptic neurons, and decreases owing to voltage leakage (see Appendix S2 for further details). When a neuron membrane potential 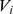 reaches a threshold value 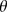 the neuron fires: 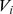 is reset to 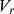 and its dynamics is switched-off during a refractory period 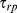. When a (pre-synaptic) neuron fires, it may open –with probability 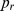
– each of the 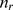 release sites existing per synapsis, inducing a current in the corresponding postsynaptic neuron. External (resp. internal) inputs, 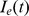 (resp. 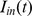) are modeled by exponentials of amplitude 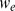 (resp. 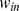) and time constant 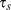. Similarly to Model A a variable 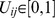 (for neuron 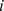 and release site 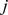) such that the release probability is modulated by 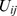, i.e. 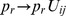, allows to implement short-time synaptic depression. 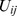 is set to 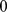 immediately after a release and recovers exponentially to 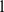 at constant rate, 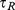 (see Appendix S2).

### Stochastic amplification of fluctuations (SAF)

Following [35] (see also [36] for an earlier reference) consider a set of deterministic equations, 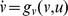 and 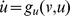, complemented respectively with additive Gaussian white noises 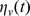 and 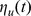, giving rise to a set of two Langevin equations. To analyze fluctuations around a fixed point 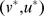 of the deterministic dynamics, a standard linear stability analysis can be performed. Defining 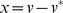 and 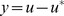, one can linearize the deterministic part of the dynamics


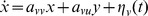



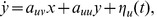
(2)

where 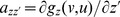 (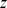 and 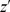 standing for either 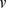 or 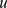) are the elements of the Jacobian matrix, 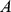, evaluated at the fixed point. The associated eigenvalues 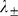 can be written as 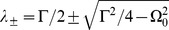 with 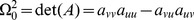 and 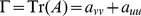.

A useful tool to identify oscillations in noisy time-series is the power spectrum 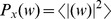, where 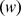 is the Fourier transform of 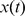 (similarly 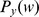 for 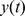), and 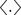 stands for independent runs average. Fourier transforming equation 2, solving for 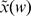 and 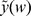, and averaging its squared modulus, we find


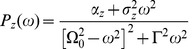
(3)

where 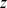 stands for 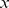 or 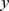, and 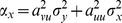, 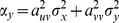. For small noise amplitudes both of the power spectra exhibit maxima near


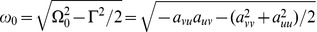
(4)

where the denominator has a minimum if 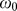 is a real number. To have a real 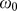 requires that both 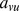 and 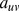 are non-vanishing and of opposite sign; when this happens, both eigenvalues of 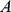 are complex (see Appendix S3). As we shall see in what follows this condition is fulfilled for Up- but not for Down states. Finally, let us underline that 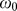 does not depend on the noise amplitude.

The presence of a non-trivial peak in the spectrum of fluctuations reflects the existence of quasi-cycles of a leading characteristic frequency, coexisting with many other frequencies, and producing a complex oscillatory pattern. Notice that, even if the peak location 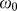 is noise independent (as long as the noise amplitude does not vanish) the very presence of a peak is a noise induced effect: in the noiseless limit the system reaches a fixed point. The phenomenon we have just described –termed stochastic amplification of fluctuations (SAF)– has been recently put forward in the context of population oscillations in Ecology [35] (see also [36]) has also been claimed to be relevant in various other areas, such as Epidemiology [37]. SAF requires the presence of some noise source acting on top of the underlying deterministic stable fixed point with complex eigenvalues 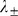, i.e. the relaxation towards the stable fixed point should be in the form of damped oscillations (this is, it is a “focus”) with a not too small damping frequency (details are explained in Appendix S3). Noise “kicks” the system away from the fixed point, and amplifies predominantly some frequency which –surprisingly enough– turns out to be *different* from the characteristic frequency of the deterministic damped oscillations (see Appendix S3). It is also noteworthy that a set of at least two coupled equations is required to have complex eigenvalues, and hence, too simplistic models in terms of only one effective variable, cannot give raise to SAF. Also, if the equations become decoupled (as it turns out to be the case for Down-states) the eigenvalues become real and the possibility of stochastic amplification is lost.

## Results

### Model A

**Figure 1:**
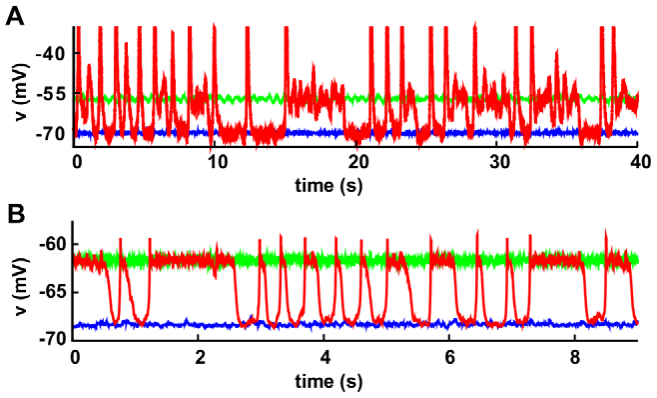
Up and Down states and Up-and-Down transitions in two different network models. (**A**) Model A (mean-field model) [18]: time-series for the membrane potential, 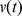. Observe the presence of two steady states lower one around 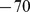 mV (Down-state/blue curve) and a larger one (Up state/green curve) at about 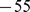 mV; these two are obtained for low noise amplitudes (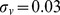 mV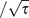, 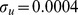 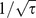) and different initial conditions. Instead, the Up-and-Down state (red curve), corresponds to a high noise amplitude (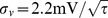, 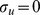). Note that, typically the Up-state intervals start with an abrupt spike which parallels empirical observations as discussed in [18]. Parameters have been fixed as in [18]: 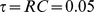 s, 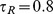 s, 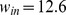 mV/Hz, 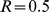, 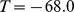 mV, 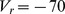 mV, and 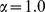 Hz/mV. (**B**) Model B (network of spiking neurons) [21]: Time series of membrane potential. Curves and color code are as for Model A. For 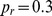 the system exhibits Up-and-Down transitions, for larger (smaller) values as 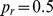 (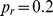), it remains steadily in the Up (Down) state. Parameters have been fixed as in [21]: vesicles per synapsis 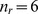, resting potential 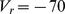 mV, membrane threshold 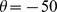 mV, capacitance 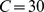 pF, leakage characteristic time 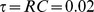 s, synaptic recovery time 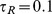 s, signal time decay 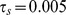 s, refractory period 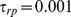 s, input amplitudes 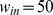 pA, 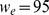 pA, and external driving rate 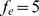 Hz.

Time-series produced by numerical simulations of such a Model A are shown in Fig. 1. Depending on the noise amplitude different outputs are produced. For low noises, either an Up state (with a high firing rate) or a stable Down state (with mean 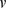 close to the resting potential, and therefore with a vanishing firing rate, and mean 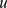 close to unity) coexist (converging into one or the other depends on the initial conditions). For larger noise Up-and-Down transitions are induced and Up-and-Down states emerge.

By performing a linear stability analysis equation 1 of as described above, we have measured the power-spectrum 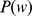, both analytically and numerically, at either the Up state and the Down state. The deterministic Up-state fixed point turns out to be a focus, with complex eigenvalues, satisfying the conditions for the existence of a non-trivial peak in the power spectra for both 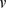 and 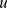. On the other hand, the Down-state fixed point (owing to the vanishing firing rate and, therefore, to the absence of crossed coupling terms (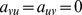 in Eq.(2)) is a node with real eigenvalues and, consequently, there is no non-trivial peak in the power-spectrum.

**Figure 2:**
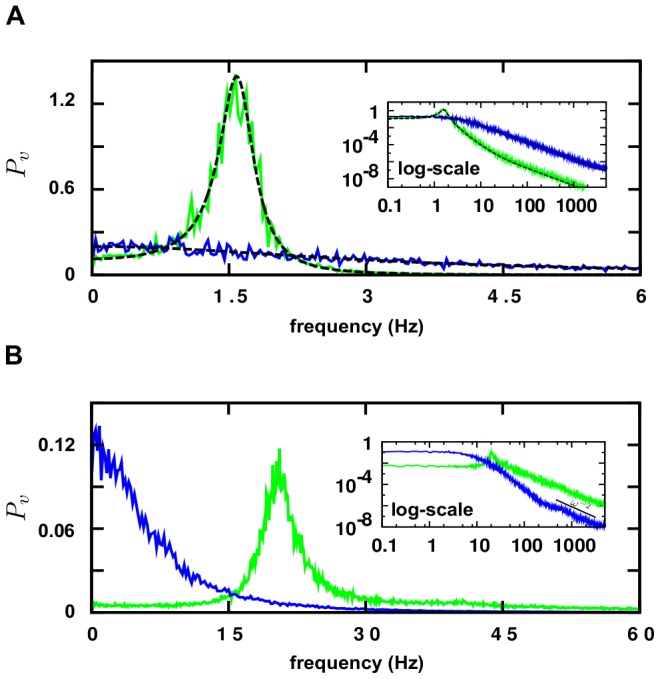
Power spectrum of membrane potential 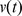 time-series in Up- and in Down states computed in Model A and Model B, respectively. Histograms are normalized to unit area. The main plots show the power-spectra in linear scale: a pronounced peak appears for the Up state (green curve) around (A) 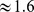 Hz and (B) 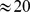 Hz. Instead, there is no track of similar peaks for Down states (blue curve). Observe the excellent agreement between simulation results (noisy curves) and analytical results for Model A, Eq.(3) (black dashed lines); for Model B a precise analytical prediction cannot be obtained. Insets represent analogous double logarithmic plots, illustrating in all cases the presence of 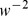 tails.

These results are illustrated in Fig. 2. Observe (**i**) the perfect agreement between analytical and numerical results in all cases, (**ii**) the presence of a peak (around 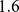 Hz) for the 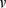 power spectrum in the Up state (note that this rhythm is much faster than that of the Up-and-Down transitions, see Fig. 1), as well as (**iii**) the absence of similar peaks for the Down-state, and finally, (**iv**) the presence of a 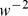 tail in all power spectra. Very similar plots can be obtained –in analogy with measurements in [26]– in the Up-intervals within Up-and-Down states as well as for 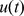 as reported in Appendix S4.

Summing up, a mean-field single-population model in presence of short-term synaptic depression as the key regulatory ingredient reproduces Up-and-Down transitions, with a non-trivial peak in the up state power spectrum emerging as a consequence of the phenomenon of SAF. Numerical results are in full agreement with this theory, and consequently no analogous peak is found in Down states.

To test the generality of this hypothesis, we have also considered the mean-field dynamics of a simple model in presence of synaptic inhibition rather than synaptic depression (cf. Appendix S1). The model also exhibits Up-Down states transitions, with a non-trivial emerging peak in the Ups but not in the Downs, consistent with SAF. Remarkably, this supports that the phenomenon of SAF invoked here remains valid beyond the particular type of neuro-physiological mechanism for network self-regulation.

Despite this success, the strategy of resorting to simplistic mean-field models presents some undeniable drawbacks: (**i**) given the lack of a detailed correspondence with neuro-physiological realistic parameters it is not possible to *quantitatively* compare the results with experimental ones; (**ii**) noise is implemented in a poorly understood way; and (**iii**) last but not least, mean-field models do not allow for comparison of individual-neuron activity with collective rhythms, which is important to figure out whether single cells frequency-lock to emergent oscillations or not. Aimed at overcoming these difficulties, in the next section we present results for a network of spiking-neurons, Model B.

### Model B

We have scrutinized Model B by numerically integrating the corresponding integrate-and-fire stochastic equations on sparse random networks as well as on regular networks. Parameters are fixed –mostly as in [21]– to neuro-biologically realistic values (see Fig. 1). We compute numerically membrane-potential and synaptic-resource time-series for each individual neuron as well as for the network as a whole. The release probability, 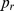, is kept as a control parameter [32]: for intermediate values as 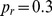 the system exhibits Up-Down transitions as illustrated in Fig. 1; for larger values (e.g. 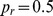) it remains steadily in the Up state, while for sufficiently low ones (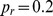) only Down states are observed (see Fig. 1).

The power-spectrum 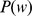 of the membrane potential time-series is illustrated in Fig. 2 (green for the Up state, blue for the Down one, both in linear and in double-logarithmic scale). Very similar plots can be obtained –in analogy with measurements in [26]– in the Up-intervals within Up-and-Down states as well as for 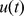 as reported in Appendix S4. In the Up state, the spectrum exhibits a sharp peak at a frequency around 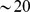 Hz, together with the expected power-law decay. On the other hand, the power spectrum for Down states lacks a similar peak. In analogy with the mean-field model in the previous section, there is a significant enhancement of the power-spectrum for Up vs Down states in the whole 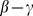 range. However, on the contrary to the model above –giving the more detailed neuron-level modeling and the use of realistic parameter values– results can be *quantitatively* compared with empirical findings. Indeed, observe that, in remarkable accordance with the experimental observations in [26] (see, e.g. Fig. 1D in [26]) the peak in the Up state spectrum lies at frequencies in the 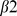-range, between 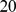 and 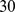 Hz. Let us remark that no parameter fine-tuning has been required to achieve this result.

Furthermore, Millman *et al.* showed in [21] that Up-and-Down states in Model B are robust against addition of fast AMPA currents, NMDA currents and (moderate) inhibition, more structured (small-world) network topologies, as well as voltage-dependent membrane resistance. Also, the non-trivial peak of the power-spectra and the associated spectral power enhancement in the 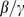 range for Up states, together with the absence of similar traits for Down states, are robust features against the extensions of the model we have scrutinized.

**Figure 3:**
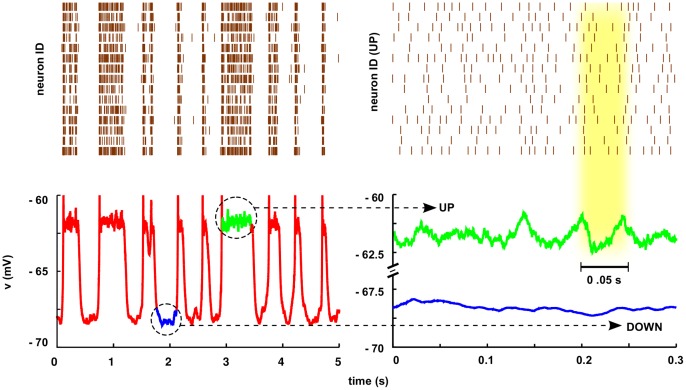
Raster plots and average membrane potential in the spiking-neuron network model (Model B). Left: (Top) Raster plot of 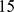 randomly chosen neurons (out of a total of 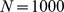 neurons in the simulation). Sticks are plotted whenever a neuron spikes. (Bottom) Time-series of the network-averaged membrane potential in the same simulation. Comparison of the two left panels (both of them sharing the same time axis) reveals that individual neurons fire often during Up states, while they are essentially quiescent in Down-state intervals. Right: (Bottom) zoom of an Up interval (green curve) and of a Down interval (blue curve); while the Up state exhibits quasi-oscillations, the Down-state does not. (Top) Raster plot of 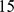 randomly chosen neurons during the Up state. Remarkably, their spiking frequency is not locked to the collective rhythm: it is about three times faster.

We have also analyzed time-series of individual neurons and compared their individual rhythms to that of the global, mean-field 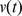. Fig. 3 (left) shows that individual neurons do follow the global trend in Up-and-Down states: global high (resp. low) average membrane potentials correspond to high (resp. low) firing rates at the individual neuron level. On the other hand, and contrary to naive expectations, within Up states (as well as within Up periods of up-and-down states) where collective quasi-oscillations for the global mean-field emerge, individual neurons do not lock themselves to such a collective rhythm; as shown in Fig. 3 (right) individual neurons fire at a much faster pace than that of the global rhythm.

Actually, a histogram of the inter-spike intervals for all neurons in the network (shown in Appendix S5) has an averaged value 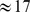 ms, corresponding to a frequency 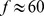 Hz. Therefore, given that the peak-frequency of the collective quasi-oscillations is located around 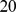 Hz each neuron fires on average 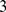 times before a cycle of the collective rhythm is completed. The same result has been achieved by analyzing the power-spectrum for individual neurons, which turns out to exhibit a peak around 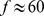 Hz and no sign of power enhancement in the 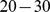 Hz band (see Appendix S5).

To firmly establish the correspondence between the just-described phenomenology for Model B and SAF we need to write down a set of effective Langevin equations, analogous to Eq.(1) for the global, network-averaged, variables and compute power-spectra from them. For a network of finite size, this can not be done in an exact way. However, as detailed in Appendix S2, the Fokker-Planck equation for the probability distribution of any *individual-neuron* membrane potential 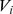 in Model B can be easily written down for infinite networks [21]. The network-averaged firing rate, 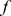, appears explicitly in such an equation, and needs to be self-consistently determined: 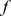 has to coincide with the outgoing probability flux, i.e. the fraction of neurons overcoming the threshold 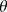 per unit time in the steady state [21]. By scrutinizing such a Fokker-Plank equation it is straightforward to see that individual neurons, follow an oscillatory pattern in which each of them is progressively charged and then fires at a pace that coincides with the (numerically determined above) rhythm of individual neurons. No track of SAF can be seen at this individual-neuron level.

In order to have an equation for the collective rhythms, we have taken the previous Fokker-Planck equation and from it computed the network-averaged membrane potential (needed to scrutinize the possible existence of SAF) at a network level, defined as


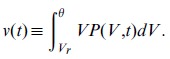
(5)

and similarly, the network-averaged synaptic depression variable 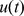. As shown in Appendix S6 they obey


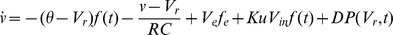



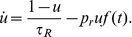
(6)

In the first equation 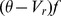 describes the average potential reduction owing to resetting, 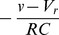 is the average leakage, 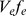 and 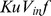 (with values of constants detailed in Appendix S2 and caption of Fig. 1) stand for the average external and internal charging, respectively, and 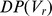 is proportional to the fraction of neurons in the resting state. The two terms in the second equation describe average recovering and consumption of synaptic resources respectively.

Eq.(6), valid for infinitely large networks, are deterministic equations. Instead, for any finite network of size 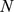, with finite connectivity and finite number of release sites, the former is no longer true: 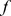 becomes a stochastic variable fluctuating around its averaged value. Something similar happens with the fraction of neurons at resting value, 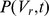 appearing in Eq.(5).

Consequently, writing 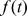 and 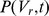 as deterministic functions (depending on both variables, 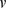 and 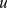) plus a noise (fluctuating part), Eq.(6) becomes a set of Langevin equations, from which power spectra could be computed. However, determining analytically the functional dependence of 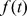 and 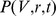 on 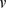 and 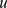 for finite values of 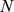 (which is necessary to perform the stability analysis) is not feasible. Owing to this, we have resorted to a numerical evaluation of such dependences. Simulation results show that 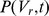 hardly departs from its infinite 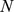 limit value, and hence its variability can be neglected for all purposes here. Instead, 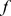 depends strongly on 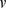 and is almost independent of 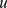; 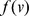 can be approximated by a “threshold-linear gain function” –zero for 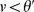 and linear when 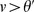
– as commonly used in the literature to approximate firing rates e.g. [18], plus a noise term, for both the Up and the Down state (see Appendix S6). It can also be verified that the amplitude of such a noise decreases with the square-root of the system size, as expected on the basis of the central limit theorem (see Appendix S6).

From 6, plugging in the approximate expression for 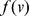 we can calculate analytically the fixed points of the deterministic dynamics, 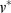 and 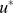. Results agree reasonably well with numerically measured averaged values both in the Up and in the Down state. Having evaluated the deterministic fixed points we can follow a standard linear stability analysis as above, compute the stability matrix, the corresponding eigenvalues, and finally the power-spectra in the Up and in the Down state as detailed above (see Appendix S6 as well as Appendix S7). For the Up state the corresponding eigenvalues turn out to be complex (i.e. as explained above, 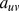 and 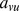 are both non-zero and of opposite signs, implying that 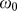 is real) entailing a non-trivial peak in the power-spectrum located at 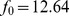 Hz. This analytical prediction slightly deviates from the numerical results as reported in Fig. 2, exhibiting a peak at 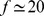 Hz. This deviation stems from the approximate nature of the present calculation. Developing a more precise analytical way to deal with finite networks remains an open and challenging task. On the other hand, for the Down state, the equations for 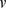 and 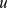 are essentially decoupled, eigenvalues are consequently real and, as a result, there is no peak in the power spectrum nor any significant enhancement of fluctuations.

In conclusion, we have shown that also for this more complex network model, an analytical (even if approximate) approach permits us to elucidate that the phenomenon of stochastic amplification of fluctuations is responsible for the non-trivial enhancement of fluctuations in the whole 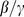 range as well as the emergence of a peak in power spectra of Up states for a frequency in the 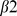 band, around 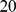 Hz. Similar results do not hold for Down states.

## Discussion

Diverse computational models –with different levels of complexity– for Up-and-Down states have been introduced in the literature. Aimed at focusing on essential aspects of the Up-Down transitions, we choose here to scrutinize models as simple as possible. In particular we have studied two different models. The first one, Model A, is a “mean-field” model defined in terms of two global variables, equipped with some additional source of stochasticity. The second, Model B, is a neuron-level based network model. Both of them are described in terms of stochastic equations for membrane potentials as well as for a second variable modeling the dynamics of synaptic depression. A mechanism of activity-dependent (short-term) synaptic depression allows the system to generate negative feedback loops, ensuing self-regulation. Under these conditions, Up and Down states and Up-and-Down transitions emerge.

We first analyzed the simpler mean-field-like Model A describing activity at a global/macroscopic level, and then went on by introducing the spiking-neuron network Model B. For these, we have first performed computer simulations, confirming the existence of Up-and-Down states. To analyze fluctuations around either the Up or the Down state, power-spectra for the global (averaged) membrane potential –which is a proxy for experimentally measured local field potentials– have been computationally measured. They show similar phenomenology in all cases: in the Up state there is a non trivial peak at some frequency together with an overall enhancement of fluctuations in the whole 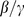 region, while no similar peak existing for Down states. These results are in excellent accordance with the experimental findings of diverse experimental groups –detailed in the Introduction– showing a similar enhancement of fluctuations under different experimental conditions in cortical Up states but never in Down states. Therefore, we conclude that existing models for Up-Down transitions succeed at reproducing realistic fluctuations in Up and Down states, as described in the Introduction.

The main contribution of the present work is to put forward that the empirically measured enhancement of fluctuations in Up states (as well as the lack of a similar effect in Down states) can be perfectly explained by the mechanism of “stochastic amplification of fluctuations”. This mechanism consists in the resonant amplification of some frequencies in the spectra of stochastic systems when the corresponding fixed-point of its deterministic dynamics is a focus (i.e. in the infinite size limit the steady state fixed point has complex associated eigenvalues). The presence of any source of noise kicks the system away from the deterministic fixed point leading to a non-trivial power-spectrum. It is important to remark that (i) empirical measurements of local field potentials correspond to mesoscopic cortex regions, intrinsically affected by noise effects and hence, a stochastic description of them is fully justified, and that (ii) curiously enough, as explained here, the selected/amplified dominant frequency is *not* that of the deterministic damped oscillations towards the focus, as it could have been naively expected.

To firmly establish the correspondence between the non-trivial features of fluctuations observed empirically as well as in computer models for Up and Down states and the phenomenon of stochastic amplification, one needs to write down a deterministic equation for the network-averaged variables and complement it with a noise term, i.e. a Langevin equation. Writing down a Langevin equation for the global dynamics of Model A, which is already a mean-field model equipped with a noise term, is a trivial task. However, this is difficult for Model B, for which we have needed to resort to a more refined approach. In both cases, we have been able to construct analytical equations (exact) for Model A and (approximate) for Model B, study the associated power-spectra, and analytically confirm the presence of non-trivial peaks appearing owing to a stochastic amplification of fluctuations for Up states (which can be described by a fixed point with complex eigenvalues at a deterministic level) but not for Down states (with real valued deterministic eigenvalues).

While for the first-studied mean-field-like Model A the agreement between experimental results and theoretical predictions is only qualitative, for the more refined spiking-neuron network Model B, the accordance becomes also quantitatively good. Indeed, observe that, in remarkable accordance with the experimental observations in [26] (see, e.g. Fig. 1D in [26]) the peak in the Up state spectrum lies at frequencies in the 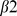-range, between 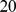 and 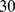 Hz.

In any case, the reported phenomenon of stochastic amplification of fluctuations explains the emergence of quasi-oscillatory –with a typical dominant frequency and a broad power-spectrum– rhythms in the global-network activity within Up states as well as (owing to the absence of a significant firing rate) the absence of a similar effect for Down states. This explanation is robust beyond modeling specificities as confirmed by the finding that many model details can be changed without affecting the results and also by the fact that a very different model, based on inhibition rather than on synaptic depression, leads to identical conclusions. Using the jargon of excitable systems, we conjecture that any activator/repressor model –the repressor being, depression, inhibition or any other form of adaptation, is in principle able to induce SAF in Up states (but not in Down states) and consequently explain the non-trivial shape of power-spectra for cortical fluctuations.

Furthermore, we have shown that the mechanism of stochastic amplification of fluctuations operates for global variables but not for individual neurons. In the framework on the neuron-level based Model B, it is possible to compare the oscillatory behavior of single neurons with the network collective rhythms. We have explicitly shown that single neurons do not lock to the global collective rhythm emerging within Up states. Actually, single neurons fire at a much faster pace –typically 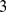 times larger– than the collective oscillation period. This phenomenology, which perfectly accounts for empirical findings in [26] as reported in the Introduction, is similar to what has been called asynchronous-states or sparse-synchronization in which a collective rhythm –to which individual neurons do *not* lock– emerges (see [38], [39] for related, though different, phenomena). Observe that in the, so-called, “fast-oscillations”, as described for instance in [38], the emerging global rhythm is much faster than individual neurons, while here it is the other way around.

In summary, Up and Down states as well as Up-and-Down transitions can be well described as collective phenomena emerging at a network level. They exhibit generically a set of highly non-trivial features which can be well captured by simple models, and perfectly accounted for by the mechanism of stochastic amplification of fluctuations.

## Supporting Information

Appendix S1 **Stochastic amplification in a excitation-inhibition mean-field model.**
(PDF)

Appendix S2 **Model B of Millman et al. and its self-consistent solution.**
(PDF)

Appendix S3 **Conditions for Stochastic amplification.**
(PDF)

Appendix S4 **Power spectrum of fluctuations for the synaptic depression variable.**
(PDF)

Appendix S5 **Characteristic frequencies for individual neuron membrane potentials.**
(PDF)

Appendix S6 **Power-spectrum evaluation for Model B.**
(PDF)

Appendix S7 **Re-scaling of the incoming currents.**
(PDF)
