# Supplementary material for: Stochastic Amplification of Fluctuations in Cortical Up-States
Source: PLoS One. 2012 Aug 7;7(8):e40710. doi: 10.1371/journal.pone.0040710 (PMC3413692; doi:10.1371/journal.pone.0040710)
Supplement: Appendix S1 — Stochastic amplification in a excitation-inhibition mean-field model. (PDF) [file pone.0040710.s001.pdf]

Appendix S1 for "Stochastic Amplification of Fluctuations in Cortical Up-states"

Jorge Hidalgo, Luís F. Seoane, Jesús M. Cortés and Miguel A. Muñoz

Departamento de Electromagnetismo y Física de la Materia and Instituto de Física Teórica y Computacional Carlos I. Facultad de Ciencias, Universidad de Granada, E-18071 Granada, Spain

Stochastic amplification in a excitation-inhibition mean-field model

Here we show that Stochastic Amplification of fluctuations can be found also in models for Up and Down states relying on populations of both, excitatory and inhibitory neurons. We consider the Wilson-Cowan-like model as described, for example, in [1] (see also [2] and [3, 4, 5] where a similar model has been recently studied). This is a mean-field like model, analogous in this sense to Model A, but with inhibition rather than depression as leading regulatory mechanism.

The model consists of two equations for the mean excitatory and inhibitory firing rates in the network:

$$\tau_e \dot{E} = -E + g(J_{ee}E - J_{ei}I + E_0) \tag{S1-1}$$
$$\tau_i \dot{I} = -I + g(J_{ie}E - J_{ii}I + I_0) \tag{S1-2}$$

with a threshold linear response function

$$g(x) = \begin{cases} 0 & x < T \\ \beta(x-T) & x \geq T \end{cases} \tag{S1-3}$$

where T is a threshold parameter. For a wide range of parameter values these equations exhibit bistability: there is a stable fixed point with with low-activity regime (Down-state) and a second one with a non-vanishing firing rate and significant activity (Up-state). Adding Gaussian white noises to both equations (S1-1) and (S1-2), the system fluctuates and eventually may jump between the two fixed points. We have verified by means of computer simulations that indeed this model exhibits Up-and-Down states, that a non-trivial peak appears for fluctuations within Up states and not for Down states. The chosen parameters are shown in Table S1.

Trajectories of the deterministic dynamics reveal spiral trajectories (i.e. damped oscillations) near the Up-state fixed point but not in the Down state (straight trajectories corresponding to real eigenvalues). Therefore, one can expect a non-trivial peak to appear in the Up-state power-spectrum but not in the Down one (see Appendix S3). This can

Parameter	Value
τ_e, τ_i	0.01 s
J_{ee}, J_{ie}, J_{ii}	5 mV/Hz
J_{ei}	9 mV/Hz
β	0.5 Hz/mV
T	15 mV
E_0	10 mV
I_0	0 mV

Table S1. Parameter values for the excitation-inhibition model presented in [1].

be explicitly seen from a linear stability analysis, and indeed

$$A_{\text{down}} = \begin{pmatrix} -\frac{1}{\tau_e} & 0 \\ 0 & -\frac{1}{\tau_i} \end{pmatrix} \tag{S1-4}$$

$$A_{\text{up}} = \begin{pmatrix} -\frac{1}{\tau_e} + \beta J_{ee} & -\beta J_{ei} \\ \beta J_{ie} & -\frac{1}{\tau_i} - \beta_{Jii} \end{pmatrix}. \tag{S1-5}$$

A_{down} is already diagonal in the Down state, with both real eigenvalues, therefore from equation (S3-5), $\omega_{0,\text{down}} \notin \mathbb{R}$ and hence, characteristic frequencies are not found in the power spectrum of fluctuations. On the other hand, eigenvalues are complex for the Up state, giving a peak in the β-range whose value is near $\omega_{0,\text{up}} = 200$ rad/s $= 31.8$ Hz. These analytical predictions are in excellent agreement with results of computer simulations for this model.

References

[1] Melamed O, Barak O, Silberberg G, Markram H, Tsodyks M (2008) Slow oscillations in neural networks with facilitating synapses. J Comput Neurosci 25: 308-316.

[2] Bressloff PC (2010) Metastable states and quasicycles in a stochastic Wilson-Cowan model of neuronal population dynamics. Phys Rev E 82: 051903.

[3] Mattia M, Giudice PD (2002) Finite-size dynamics of inhibitory and excitatory interacting spiking neurons. Phys Rev E 66: 051917.

[4] Mattia M, Giudice PD (2004) Finite-size dynamics of inhibitory and excitatory interacting spiking neurons. Phys Rev E 70: 052903.

[5] Wallace E, Benayoun M, van Drongelen W, Cowan JD (2011) Emergent oscillations in networks of stochastic spiking neurons. PLoS ONE 6: e14804.
