# Supplementary material for: Stochastic Amplification of Fluctuations in Cortical Up-States
Source: PLoS One. 2012 Aug 7;7(8):e40710. doi: 10.1371/journal.pone.0040710 (PMC3413692; doi:10.1371/journal.pone.0040710)
Supplement: Appendix S2 — Model B of Millman et al. and its self-consistent solution. (PDF) [file pone.0040710.s002.pdf]

Appendix S2 for "Stochastic Amplification of Fluctuations in Cortical Up-states"

Jorge Hidalgo, Luís F. Seoane, Jesús M. Cortés and Miguel A. Muñoz

Departamento de Electromagnetismo y Física de la Materia and Instituto de Física Teórica y Computacional Carlos I. Facultad de Ciencias, Universidad de Granada, E-18071 Granada, Spain

Model B of Millman et al. and its self-consistent solution

The model studied by Millman *et al.*, Model B, is defined by the following set of equations for the membrane potential V_i of neuron i and the synaptic utility U_{ij} for each release site j:

$$\begin{aligned}\dot{V}_i =& -\frac{V_i - V_r}{RC} + \frac{1}{C}\sum_k I^k_{\mathrm{e}_i}(t) + \\ & \frac{1}{C}\sum_{\substack{i'j' \\ \text{linking } i}} \sum_k \Theta(p_r U_{i'j'}(t^k_{s_{i'}}) - \zeta^k_{i'j'}) I^k_{\mathrm{in}_{i'}}(t), \\ \dot{U}_{ij} =& \frac{1-U_{ij}}{\tau_R} - \sum_k U_{ij}\Theta(p_r - \zeta^k_{ij})\delta(t - t^k_{s_i}). \end{aligned} \tag{S2-1}$$

where ζ^k_{ij} is a uniform random number in $[0,1]$ and $\Theta(x)$ the Heaviside step function. The first term in the r.h.s. of the first equation describes the leakage, the second is the sum over external inputs (Poisson distributed at rate f_e), and the third represents the internal currents arriving from (pre-synaptic) neuron i' to (post-synaptic) neuron i at every release site j'; there are n_r release sites per synapsis; k runs over spikes, occurring at times $t^k_{s_i}$ for each neuron i.

The Fokker-Planck equation proposed in [1] to describe this model in the limit of infinitely large system-size is

$$\begin{aligned}\frac{\partial P(V,t)}{\partial t} =& -\frac{\partial F(V,t)}{\partial V} = \frac{\partial[\nu_d(V,t)P(V,t)]}{\partial V} + D\frac{\partial^2 P(V,t)}{\partial V^2} \\ =& \frac{\partial\left[(-\frac{V-V_r}{RC} + V_{\mathrm{e}}f_e + KuV_{\mathrm{in}}f)P(V,t)\right]}{\partial V} + \frac{1}{2}\left(V_{\mathrm{e}}^2 f_e + Ku^2V_{\mathrm{in}}^2 f\right)\frac{\partial^2 P(V,t)}{\partial^2 V}.\end{aligned} \tag{S2-2}$$

The drift (or deterministic) term in equation (S2-2) includes potential leakage and external plus internal input integration. The diffusion term stems from the Poisson-like nature assumed for both external and internal spikes (K synapses per neuron; i.e. finite

connectivity). f stands for averaged firing rate and $V_{\text{in}} = p_r n_r w_{\text{in}} \tau_s / C$ and $V_{\text{e}} = w_{\text{e}} \tau_s / C$ are the mean increase in membrane potential from a single internal and external (exponential) input. Indeed, observe that the factor τ_s in the expressions $V_{\text{in/e}}$ comes from $\int_{t_s}^{\infty} e^{-(t-t_s)/\tau_s} dt = \tau_s$. In order to enhance the accuracy of the quantitative agreement between theoretical predictions and numerical results, we have improved this estimation of the global membrane potential increase per spike by taking into account that neurons are eventually reset and during their refractory period they do not integrate stimuli and the arriving inputs are interrupted. In this way, (see S7) the average input per spike becomes

$$\overline{V}_{\text{in/e}} = V_{\text{in/e}} \left[1 - f\tau_s \left(1 - e^{-\frac{1}{f\tau_s}} \right) \right], \tag{S2-3}$$

which represents a significant change with respect to $V_{\text{in/e}}$ above.

Some remarks are in order:

- In the fully connected case $K = N$, assuming that internal input amplitudes are rescaled by the average connectivity (i.e. $w_{\text{in}} \to w_{\text{in}}/K$) in order to keep the total signal per spike constant, the internal noise disappears in the infinite size limit. In other words, the internal contribution to the diffusion term, proportional to V_{in} stems from the finite connectivity of each individual neuron in sparse networks. Similarly, in the absence of external stochasticity, the external contribution to the diffusion term, proportional to V_{e} would disappear for a homogeneously distributed excitation. If the two previous conditions hold, the dynamics becomes purely deterministic.

- Observe that only derivatives with respect to V, and not u, appear in equation (S2-2); this is because, the synaptic depression variable has been averaged also over release-sites, hence, in the limit of large n_r it is replaced by its mean-field value which obeys

$$\dot{u}(t) = \frac{1-u}{\tau_R} - p_r f u. \tag{S2-4}$$

  This replacement is accurate for large values of n_r while otherwise it is just an approximation.

Equation (S2-2) needs to be complemented with the boundary conditions $F(V_r, t + \tau_{rp}) = F(\theta, t)$ where $F(V,t) = (V_{\text{e}}^2 f_e + K u^2 V_{\text{in}}^2 f) \frac{\partial P(V,t)}{\partial V}$ is the flux at a given value of V, and $P(\theta, t) = 0$, representing the fact that neurons at threshold are instantly reset to the resting-potential V_r, and kept inactive for a refractory period τ_{rp} [2, 1]. The firing rate, f, is computed as the outgoing probability flux, i.e. the fraction of neurons overcoming θ per unit time, $f(t) = F(\theta, t)$.

As the dynamics depends on the probability flux f, which on its turn is fixed by the overall dynamics, the Fokker-Planck equation needs to be solved self-consistently. This can be done numerically (Euler-implicit method) giving results in agreement with those in [1]: there are two different stable states for the probability distribution (see figure S2).

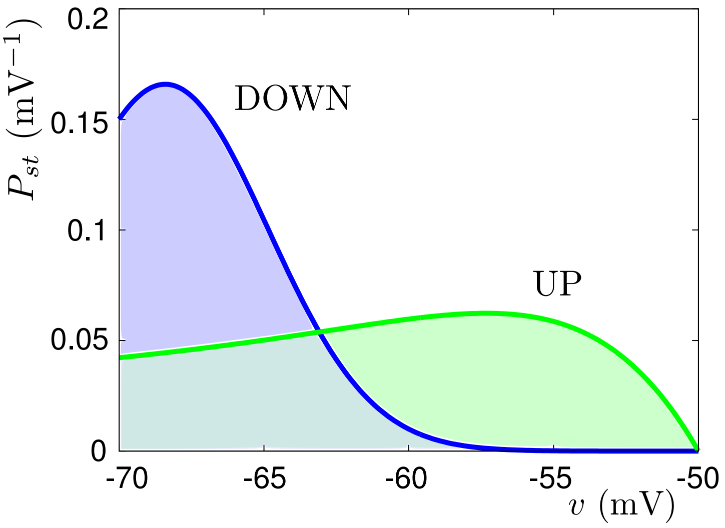

Figure S2. Solutions for the membrane potential distributions described by equation (S2-2). In the Down-state, membrane potentials are closer to V_r, and the slope in θ gives a low firing rate $f = 0.00022$ Hz, while in the Up-state, potentials raise up, giving $f = 74.9$ Hz.

References

[1] Millman D, Mihalas S, Kirkwood A, Niebur E (2010) Self-organized criticality occurs in non-conservative neuronal networks during 'up' states. Nat Phys 6: 801-805.

[2] Brunel N, Hansel D (2006) How noise affects the synchronization properties of recurrent networks of inhibitory neurons. Neural Comput 18: 1066-1110.
