# Supplementary material for: Stochastic Amplification of Fluctuations in Cortical Up-States
Source: PLoS One. 2012 Aug 7;7(8):e40710. doi: 10.1371/journal.pone.0040710 (PMC3413692; doi:10.1371/journal.pone.0040710)
Supplement: Appendix S3 — Conditions for Stochastic amplification. (PDF) [file pone.0040710.s003.pdf]

Appendix S3 for "Stochastic Amplification of Fluctuations in Cortical Up-states"

Jorge Hidalgo, Luís F. Seoane, Jesús M. Cortés and Miguel A. Muñoz

Departamento de Electromagnetismo y Física de la Materia and Instituto de Física Teórica y Computacional Carlos I. Facultad de Ciencias, Universidad de Granada, E-18071 Granada, Spain

Conditions for Stochastic amplification

Let us consider the stability (Jacobian) matrix, A, of a two-variable system and let $\lambda_\pm$ be its associated eigenvalues. In general, they can be written as complex numbers

$$\lambda_\pm = \lambda_\pm^R + i\lambda_\pm^I. \tag{S3-1}$$

As A is a real matrix, its determinant and its trace are both real. This imposes some constraints on the eigenvalues: $\mathrm{Tr}(A) = \lambda_+^R + \lambda_-^R + i(\lambda_+^I + \lambda_-^I) \in \mathbb{R}$ and hence

$$\lambda_+^I = -\lambda_-^I \equiv \lambda^I. \tag{S3-2}$$

Similarly, $\det(A) = \lambda_+^R\lambda_-^R - \lambda_+^I\lambda_-^I + i(\lambda_+^R\lambda_-^I + \lambda_-^R\lambda_+^I) \in \mathbb{R}$, and therefore

$$\lambda_+^R = \lambda_-^R \equiv \lambda^R \quad \text{if} \quad \lambda^I \neq 0. \tag{S3-3}$$

As shown in the main text the power-spectrum can be expressed as

$$P(\omega) = \frac{\alpha_z + \sigma_z^2\omega^2}{[\mathrm{Det}(A) - \omega^2]^2 + (\mathrm{Tr}A)^2\omega^2}, \tag{S3-4}$$

which has a maximum around

$$\omega_0 = \sqrt{\det(A) - (\mathrm{Tr}A)^2/2} = \sqrt{-\frac{1}{2}(\lambda_+^2 + \lambda_-^2)} \tag{S3-5}$$

where the denominator vanishes, provided that ω_0 is real. Taking into account equations (S3-2) and (S3-3),

$$\omega_0 = \sqrt{(\lambda^I)^2 - (\lambda^R)^2} \tag{S3-6}$$

which provides a direct way to compute ω_0. In particular, observe that $|\lambda^I| > |\lambda^R|$ is a necessary and sufficient condition for a non-trivial maximum to exist, and hence, the system does not exhibit Stochastic amplification if λ^I is zero or not sufficiently large. Notice that, if A is diagonal (i.e. the two equations become decoupled), $\lambda^I = 0$ and no stochastic amplification can occur. Indeed, it suffices that only one of the non-diagonal terms of A is zero to rule out stochastic amplification.

Stochastic amplification of fluctuations occurs when the deterministic system falls with damped oscillations (spiral decay towards the focus, as corresponds to complex eigenvalues). Noise perturbs trajectories, kicking them away from the focus and sustaining oscillations. It is noteworthy that the selected oscillation frequency does *not* coincide with that of the transitory deterministic dynamics, $\omega^* = |\lambda^I| = \sqrt{\det(A) - \mathrm{Tr}(A)^2/4}$, i.e. $\omega_0 \neq \omega^*$.
