# Supplementary material for: Stochastic Amplification of Fluctuations in Cortical Up-States
Source: PLoS One. 2012 Aug 7;7(8):e40710. doi: 10.1371/journal.pone.0040710 (PMC3413692; doi:10.1371/journal.pone.0040710)
Supplement: Appendix S4 — Power spectrum of fluctuations for the synaptic depression variable. (PDF) [file pone.0040710.s004.pdf]

Appendix S4 for "Stochastic Amplification of Fluctuations in Cortical Up-states"

Jorge Hidalgo, Luís F. Seoane, Jesús M. Cortés and Miguel A. Muñoz

Departamento de Electromagnetismo y Física de la Materia and Instituto de Física Teórica y Computacional Carlos I. Facultad de Ciencias, Universidad de Granada, E-18071 Granada, Spain

Power spectrum of fluctuations for the synaptic depression variable

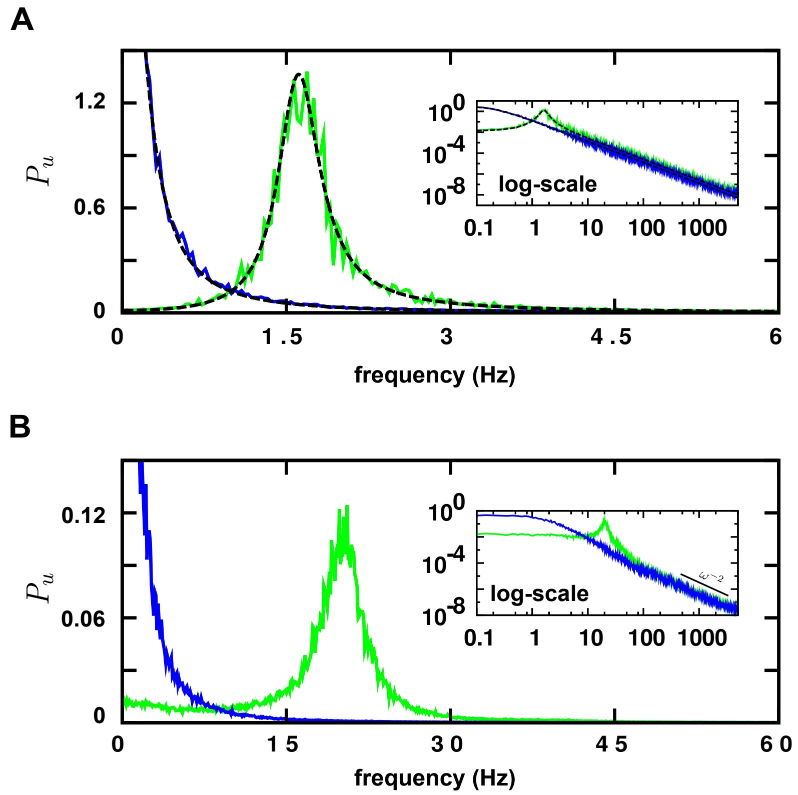

Figure S4. Power spectrum for the variable synaptic depression variable, u, in Up and in Down states for (A) Model A and (B) Model B, respectively. Plots are normalized to unit area. As in Fig. 2 of main text, similar peaks appear for Up (green curves) but not for Down (blue curves): (A) $\approx$ 1.6 Hz and (B) $\approx$ 20 Hz. Insets present power spectra in double logarithmic scale; spectra exhibit a ω^{-2} tail indicating the presence of many different scales.
