# Supplementary material for: Stochastic Amplification of Fluctuations in Cortical Up-States
Source: PLoS One. 2012 Aug 7;7(8):e40710. doi: 10.1371/journal.pone.0040710 (PMC3413692; doi:10.1371/journal.pone.0040710)
Supplement: Appendix S5 — Characteristic frequencies for individual neuron membrane potentials. (PDF) [file pone.0040710.s005.pdf]

Appendix S5 for "Stochastic Amplification of Fluctuations in Cortical Up-states"

Jorge Hidalgo, Luís F. Seoane, Jesús M. Cortés and Miguel A. Muñoz

Departamento de Electromagnetismo y Física de la Materia and Instituto de Física Teórica y Computacional Carlos I. Facultad de Ciencias, Universidad de Granada, E-18071 Granada, Spain

Characteristic frequencies for individual neuron membrane potentials

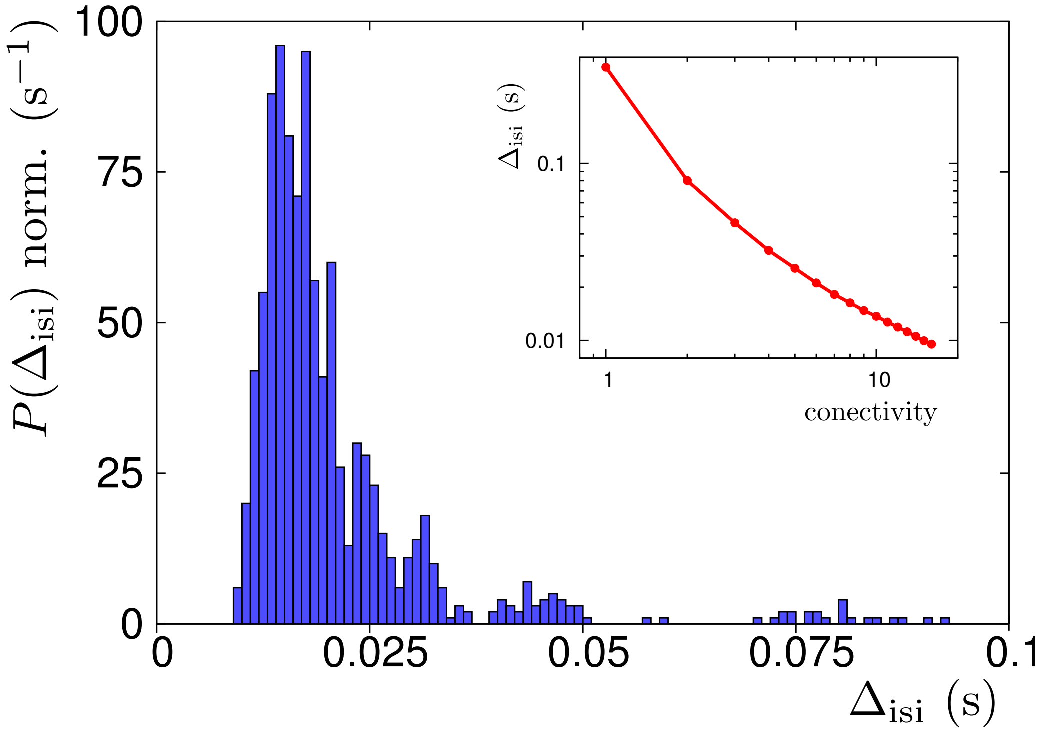

Figure S5-1. Probability distribution of the inter-spike-intervals in the random network; its average gives $\langle\Delta_{isi}\rangle \approx 17$ ms, corresponding to a mean firing rate of $f \approx 60$ Hz. This result perfectly agrees with mean firing rate of Fig. S5-2. Heterogeneity in the average inter-spike-intervals stems from the different connectivity degrees, as illustrated in the inset. In the latter, we show the average value of Δ_{isi} for different (pre-synaptic) connectivity levels, exhibiting a $\sim 1/K$ dependency.

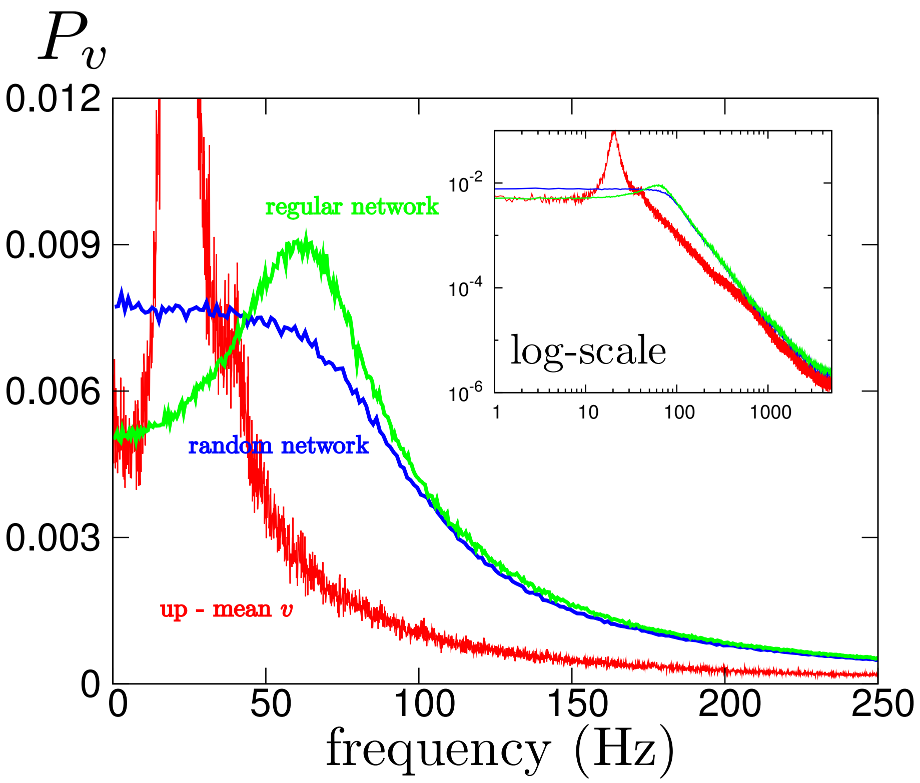

Figure S5-2. Averaged power spectra for individual neuron membrane-potential time-series for both a random network with average connectivity $K = 7.5$ (blue) and a regular network with connectivity $K = 7$ (green) in the Up state. A sharp peak (around 60 Hz) is seen for regular networks; instead in random networks the peak is blurred owing to node-to-node heterogeneity. In any case, there is no peak at the characteristic frequency of the global, network-averaged membrane potential, ≈ 20 Hz (peak of the red curve): individual neurons do not lock to the collective rhythm within Up states. The inset shows a similar plot in logarithmic scale, putting forward the presence of a distinct peak for regular networks together with a w^{-2} tail for all spectra.
