# Supplementary material for: Stochastic Amplification of Fluctuations in Cortical Up-States
Source: PLoS One. 2012 Aug 7;7(8):e40710. doi: 10.1371/journal.pone.0040710 (PMC3413692; doi:10.1371/journal.pone.0040710)
Supplement: Appendix S6 — Power-spectrum evaluation for Model B. (PDF) [file pone.0040710.s006.pdf]

Appendix S6 for "Stochastic Amplification of Fluctuations in Cortical Up-states"

Jorge Hidalgo, Luís F. Seoane, Jesús M. Cortés and Miguel A. Muñoz

Departamento de Electromagnetismo y Física de la Materia and Instituto de Física Teórica y Computacional Carlos I. Facultad de Ciencias, Universidad de Granada, E-18071 Granada, Spain

Power-spectrum evaluation for Model B

To firmly establish the correspondence between the phenomenology described for Model B in the main text and stochastic amplification we need to write down a set of effective Langevin equations (analogous to equation 1 in the main text) for the network averaged variables and, from it, compute power-spectra. This turns out to be a non-trivial task.

Our starting point is equation (S2-2). Multiplying it by V and integrating over all possible values of the membrane potential variable

$$\begin{aligned}
\dot{v}(t) = & \int_{V_r}^{\theta} V \frac{\partial P(V,t)}{\partial t} dV = VD\frac{\partial P(V,t)}{\partial V}\bigg|_{V_r}^{\theta} - D\int_{V_r}^{\theta} \frac{\partial P(V,t)}{\partial V} dV \\
& - V\nu_d(V)P(V,t)\bigg|_{V_r}^{\theta} + \int_{V_r}^{\theta} \nu_d(V)P(V,t)dV \\
= & \, \theta D\frac{\partial P(\theta,t)}{\partial V} - V_r D\frac{\partial P(V_r,t)}{\partial V} + DP(V_r,t) + V_r\nu_d(V_r)P(V_r,t) + \int_{V_r}^{\theta} \nu_d(V)P(V,t)dV \\
= & -(\theta - V_r)f(t) - \frac{v - V_r}{RC} + V_e f_e + KuV_{\text{in}}f(t) + DP(V_r,t), \qquad \text{(S6-1)}
\end{aligned}$$

where boundary conditions have been imposed and τ_{rp} has been, for simplicity, neglected.

The self-consistent method used for solving equation (S2-2) together with equation (S2-4) provides v^*, u^*, f^* and $P(V_r)^*$, computed via the mean, the slope in θ and the value in V_r of the steady state solution $P(V)$. Results are shown in Table S6. Differences with simulation results ($\langle v\rangle_{\text{up}} = -61.67\,\text{mV}, \langle u\rangle_{\text{up}} = 0.2352; \langle v\rangle_{\text{down}} = -68.3\,\text{mV}, \langle u\rangle_{\text{down}} = 0.997$) stem from the relatively small value $n_r = 6$ used in simulations; as explained above, the Fokker-Planck approach is strictly valid for $n_r \to \infty$.

In the case of finite networks, $P(V_r,t)$ and $f(t)$ become fluctuating time-dependent variables. Fig. S6-1 illustrates the result of numerical simulations for network of various sizes for the firing rate: $f(t)$ is observed to be strongly correlated with $v(t)$; the larger the average potential the larger the fraction of neurons firing per unit time. The inset of Fig. S6-1, where f is plotted as a function of v and u, illustrates the existence of two well-defined branches, one for up-to-down transitions and another for down-to-up, when f is considered a function of v.

	**Up** ($p_r = 0.5$)	**Down** ($p_r = 0.2$)
v^* (mV)	-61.16	-66.54
u^*	0.2108	0.999996
f^* (Hz)	74.88	0.000216
$P(V_r)^*$ (V^{-1})	40.75	150.04

Table S6. Results obtained from the Up and Down steady state distributions shown in Fig. S2.

For the forthcoming analytical calculations, f can be well approximated by a threshold-linear (or "split") function of v plus a noise, and with this we describe its shape in both Up and Down states. The noise amplitude, as shown in Fig. S6-2 decreases with network-size as expected on the central limit theorem basis.

As for the probability density of neurons at the resting state, $P(V_r, t)$, it is consider as a constant of value $P(V_r)^*$ for simplicity.

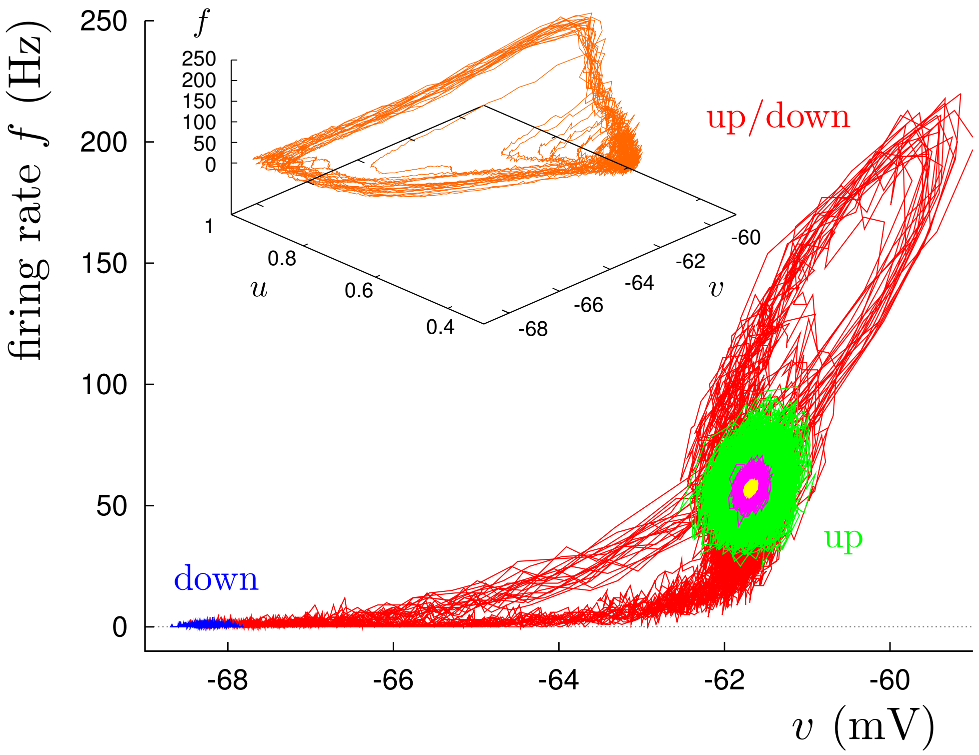

Figure S6-1. Main: Firing rate f as a function of the mean membrane potential v for various finite-size networks in the model of Millman *et al.* for (red) the Up-and-Down state $p_r = 0.3$ and $N = 10^3$, (blue) Down-state $p_r = 0.2$ and $N = 10^3$, and (green $N = 10^3$, magenta 10^4, and yellow $N = 10^5$) Up-state $p_r = 0.5$. By increasing the system size the cloud of points converges to the steady state fixed point. The approximate linear fit is: $f_{\rm up}(v) = (12.86 \pm 0.05\,{\rm Hz/mV})v + (850 \pm 3){\rm Hz}$ for the Up-state and $f(v) = 0$ for the Down-state. Even in the case of Up-and-Down states, $f(v)$ can be well approximated by a bi-valuated function with two branches: one for transitions Down-to-Up and other for Up-to-Down. Inset: f as a function of both v and u illustrating the origin of the two branches above.

Having an analytical approximation for $f(v)$ it is now possible to perform a lineal

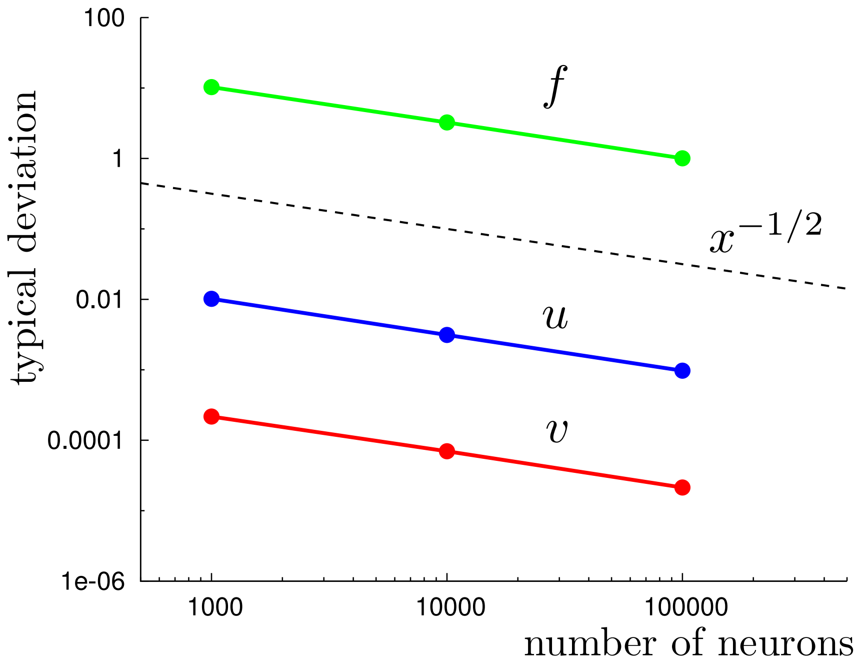

Figure S6-2. Typical deviation of fluctuations for different variables as a function of the system size. Simulations -computed for a persistent Up state– show a decay of $1/\sqrt{N}$, as expected on the basis of the central limit theorem.

stability analysis. Defining $x = v - v^*$ and $y = u - u^*$ as the linear deviations from the deterministic fixed points, the corresponding Jacobian matrix is specified as follows:

$$\begin{aligned}
a_{vv} &= -\frac{1}{RC} + f' \left\{ n_s u \bar{V}_{in} \left(1 + \frac{1}{2} u \bar{V}_{in} P(V_r) \right) - (\theta - V_r) \right\} \\
&\quad + G \left\{ \bar{V}_e f_e + n_s f u \bar{V}_{in} + 2DP(V_r) \right\} \\
a_{vu} &= n_s \bar{V}_{in} f \left\{ 1 + u \bar{V}_{in} P(V_r) \right\} \\
a_{uv} &= -u p_r f' \\
a_{uu} &= -\frac{1}{\tau_R} - p_r f
\end{aligned} \tag{S6-2}$$

where G is the derivative of the re-scaling factor of the incoming currents (see S7) which depends on $f(v)$:

$$G \equiv \frac{\tau_s f' \left\{ e^{-\frac{1}{f\tau_s}} \left(1 + \frac{1}{\tau_s f} \right) - 1 \right\}}{1 - f\tau_s \left\{ 1 - e^{-\frac{1}{f\tau_s}} \right\}} \tag{S6-3}$$

giving a non-trivial correction.

At the Up-state fixed point this leads to $a_{vv} = -120.12$ Hz, $a_{vu} = 10.4272$ V·Hz, $a_{uv} = -1355.44$ Hz/V, $a_{uu} = -47.4422$ Hz for the coefficients of the stability matrix, and hence a minimum at the denominator of $P(w)$ at $w_0 = 76.1$ rad/s $\Longrightarrow f_0 = 12.11$ Hz. Instead, in the Down-state, the equation for u becomes decoupled from that for v, resulting in the absence of a non-trivial peak in the spectrum (complex ω_0), even for a small but non zero firing rate.
