# Supplementary material for: Stochastic Amplification of Fluctuations in Cortical Up-States
Source: PLoS One. 2012 Aug 7;7(8):e40710. doi: 10.1371/journal.pone.0040710 (PMC3413692; doi:10.1371/journal.pone.0040710)
Supplement: Appendix S7 — Re-scaling of the incoming currents. (PDF) [file pone.0040710.s007.pdf]

Appendix S7 for "Stochastic Amplification of Fluctuations in Cortical Up-states"

Jorge Hidalgo, Luís F. Seoane, Jesús M. Cortés and Miguel A. Muñoz

Departamento de Electromagnetismo y Física de la Materia and Instituto de Física Teórica y Computacional Carlos I. Facultad de Ciencias, Universidad de Granada, E-18071 Granada, Spain

Re-scaling of the incoming currents

When a neuron fires, it is kept silent during the refractory period and it "ignores" all the arriving currents. As the tails of exponential (internal or external) currents can be interrupted by this mechanism, taking the mean value of the exponential function (integrated between 0 and ∞) is not a good approximation. A better estimation can be obtained as follows. In the mean-field approach, a neuron fires every $1/f$ seconds; meanwhile, the incoming currents contribute to increment its membrane potential; a schematic representation is shown in Fig. S7.

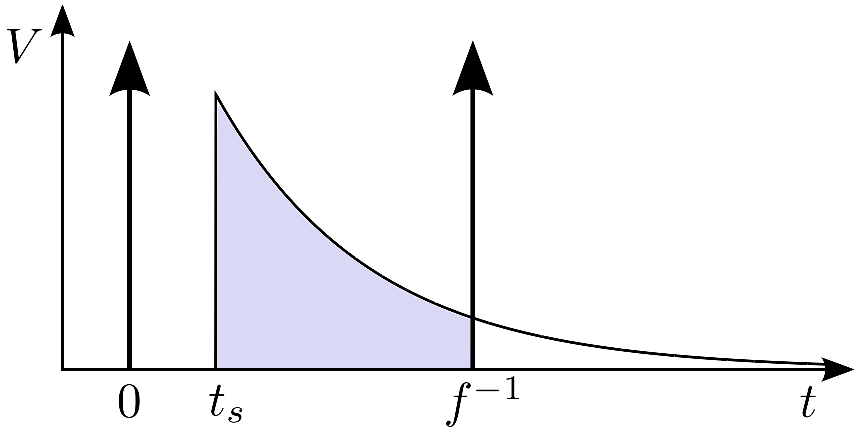

Figure S7. Representation of an incoming, exponential current integrating until the post-synaptic neuron fires, entering into the refractory period. Losses depend on the spiking time of the pre-synaptic neuron and the firing rate of the post-synaptic one.

Therefore, one can consider that the neuron integrates on average from the spiking-time $t = 0$ to some effective final time $t = 1/f$. The contribution of an incoming spike arriving at $t = t_s$ can be computed as

$$\overline{V}_{\mathrm{e/in}}(t_s) = \int_{t_s}^{f^{-1}} w_{\mathrm{e/in}} e^{-\frac{t-t_s}{\tau_s}} dt = V_{\mathrm{e/in}} \left[1 - e^{-\frac{f^{-1}-t_s}{\tau_s}} \right], \tag{S7-1}$$

where $V_{\mathrm{e/in}} = w_{\mathrm{e/in}}\tau_s/C$. As many spikes arrive during the interval $[0, 1/f]$, supposing an uniform distribution for the incoming times, the mean value of this t_s-function is

$$\bar{V}_{\mathrm{e/in}} \equiv \frac{\displaystyle\int_0^{f^{-1}} \bar{V}_{\mathrm{e/in}}(t_s)dt_s}{f^{-1}} = V_{\mathrm{e/in}}\left[1 - f\tau_s\left(1 - e^{-\frac{1}{f\tau_s}}\right)\right]. \tag{S7-2}$$

Observe that an extra factor multiplying the mean value of the exponential has appeared with respect to the naive estimation in [1]. For typical values of $f \approx 100$ Hz and $\tau_s = 5$ ms, the mean contribution of incoming spikes is rescaled by a more than a 50%.

References

[1] Millman D, Mihalas S, Kirkwood A, Niebur E (2010) Self-organized criticality occurs in non-conservative neuronal networks during 'up' states. Nat Phys 6: 801-805.
